# First trimester maternal tryptophan metabolism and embryonic and fetal growth: the Rotterdam Periconceptional Cohort (Predict Study)

**DOI:** 10.1093/humrep/deae046

**Published:** 2024-03-18

**Authors:** Sofie K M van Zundert, Nina C M van Egmond, Lenie van Rossem, Sten P Willemsen, Pieter H Griffioen, Ron H N van Schaik, Mina Mirzaian, Régine P M Steegers-Theunissen

**Affiliations:** Department of Obstetrics and Gynecology, Erasmus MC, University Medical Center, Rotterdam, the Netherlands; Department of Clinical Chemistry, Erasmus MC, University Medical Center, Rotterdam, the Netherlands; Department of Obstetrics and Gynecology, Erasmus MC, University Medical Center, Rotterdam, the Netherlands; Department of Clinical Chemistry, Erasmus MC, University Medical Center, Rotterdam, the Netherlands; Department of Obstetrics and Gynecology, Erasmus MC, University Medical Center, Rotterdam, the Netherlands; Department of Obstetrics and Gynecology, Erasmus MC, University Medical Center, Rotterdam, the Netherlands; Department of Biostatistics, Erasmus MC, University Medical Center, Rotterdam, the Netherlands; Department of Clinical Chemistry, Erasmus MC, University Medical Center, Rotterdam, the Netherlands; Department of Clinical Chemistry, Erasmus MC, University Medical Center, Rotterdam, the Netherlands; Department of Clinical Chemistry, Erasmus MC, University Medical Center, Rotterdam, the Netherlands; Department of Obstetrics and Gynecology, Erasmus MC, University Medical Center, Rotterdam, the Netherlands

**Keywords:** tryptophan metabolism, 5-hydroxytryptophan, kynurenine, periconception period, embryonic growth

## Abstract

**STUDY QUESTION:**

What is the association between first trimester maternal tryptophan (TRP) metabolites and embryonic and fetal growth?

**SUMMARY ANSWER:**

Higher 5-hydroxytryptophan (5-HTP) concentrations are associated with reduced embryonic growth and fetal growth and with an increased risk of small-for-gestational age (SGA), while higher kynurenine (KYN) concentrations are associated with a reduced risk of SGA.

**WHAT IS KNOWN ALREADY:**

The maternal TRP metabolism is involved in many critical processes for embryonic and fetal growth, including immune modulation and regulation of vascular tone. Disturbances in TRP metabolism are associated with adverse maternal and fetal outcomes.

**STUDY DESIGN, SIZE, DURATION:**

This study was embedded within the Rotterdam Periconceptional Cohort (Predict Study), an ongoing prospective observational cohort conducted at a tertiary hospital from November 2010 onwards.

**PARTICIPANTS/MATERIALS, SETTING, METHODS:**

A total of 1115 women were included before 11 weeks of gestation between November 2010 and December 2020. Maternal serum samples were collected between 7 and 11 weeks of gestation, and TRP metabolites (TRP, KYN, 5-HTP, 5-hydroxytryptamine, and 5-hydroxyindoleacetic acid) were determined using a validated liquid chromatography (tandem) mass spectrometry method. Serial 3D ultrasound scans were performed at 7, 9, and 11 weeks of gestation to accurately assess features of embryonic growth, including crown–rump length (CRL) and embryonic volume (EV) offline using virtual reality systems. Fetal growth parameters were retrieved from medical records and standardized according to Dutch reference curves. Mixed models were used to assess associations between maternal TRP metabolites and CRL and EV trajectories. Linear and logistic regression models were utilized to investigate associations with estimated fetal weight (EFW) and birthweight, and with SGA, respectively. All analyses were adjusted for potential confounders.

**MAIN RESULTS AND THE ROLE OF CHANCE:**

Maternal 5-HTP concentrations and the maternal 5-HTP/TRP ratio were inversely associated with embryonic growth (5-HTP, √CRL: β = –0.015, 95% CI = –0.028 to –0.001; 5-HTP ^3^√EV: β = –0.009, 95% CI = –0.016 to –0.003). An increased maternal 5-HTP/TRP ratio was also associated with lower EFW and birthweight, and with an increased risk of SGA (odds ratio (OR) = 1.006, 95% CI = 1.00–1.013). In contrast, higher maternal KYN concentrations were associated with a reduced risk of SGA in the unadjusted models (OR = 0.548, 95% CI = 0.320–0.921).

**LIMITATIONS, REASONS FOR CAUTION:**

Residual confounding cannot be ruled out because of the observational design of this study. Moreover, this study was conducted in a single tertiary hospital, which assures high internal validity but may limit external validity.

**WIDER IMPLICATIONS OF THE FINDINGS:**

The novel finding that maternal 5-HTP concentrations are associated with a smaller embryo and fetus implies that disturbances of the maternal serotonin pathway in the first trimester of pregnancy are potentially involved in the pathophysiology of fetal growth restriction. The association between higher maternal KYN concentrations and a reduced risk of SGA substantiate the evidence that the KYN pathway has an important role in fetal growth. More research is needed to delve deeper into the potential role of the maternal TRP metabolism during the periconception period and pregnancy outcome for mother and offspring.

**STUDY FUNDING/COMPETING INTEREST(S):**

This study was funded by the Department of Obstetrics and Gynecology and the Department of Clinical Chemistry of the Erasmus MC, University Medical Center, Rotterdam, the Netherlands. The authors have no competing interests to disclose.

**TRIAL REGISTRATION NUMBER:**

N/A.

## Introduction

Embryonic and fetal growth are important determinants for birth outcomes and health later in life. Especially during the periconception period, when critical processes occur and growth rates are the highest, maternal exposures can have profound effects on growth of the embryo (Dickey and Gasser, 1993; Gluckman *et al.*, 2010; Steegers-Theunissen *et al.*, 2013). Indeed, maternal exposures, such as a poor diet quality and smoking, during the periconception period have been found to reduce embryonic growth (van Uitert *et al.*, 2013b; Abdollahi *et al.*, 2021; Pietersma *et al.*, 2022). Previous studies have revealed that smaller embryos are associated with reduced fetal growth, lower birthweight, and cardiovascular risk factors in the future child (Mook-Kanamori *et al.*, 2010; Jaddoe *et al.*, 2014; Ustunyurt *et al.*, 2015; Xu *et al.*, 2022).

**Figure 1.**
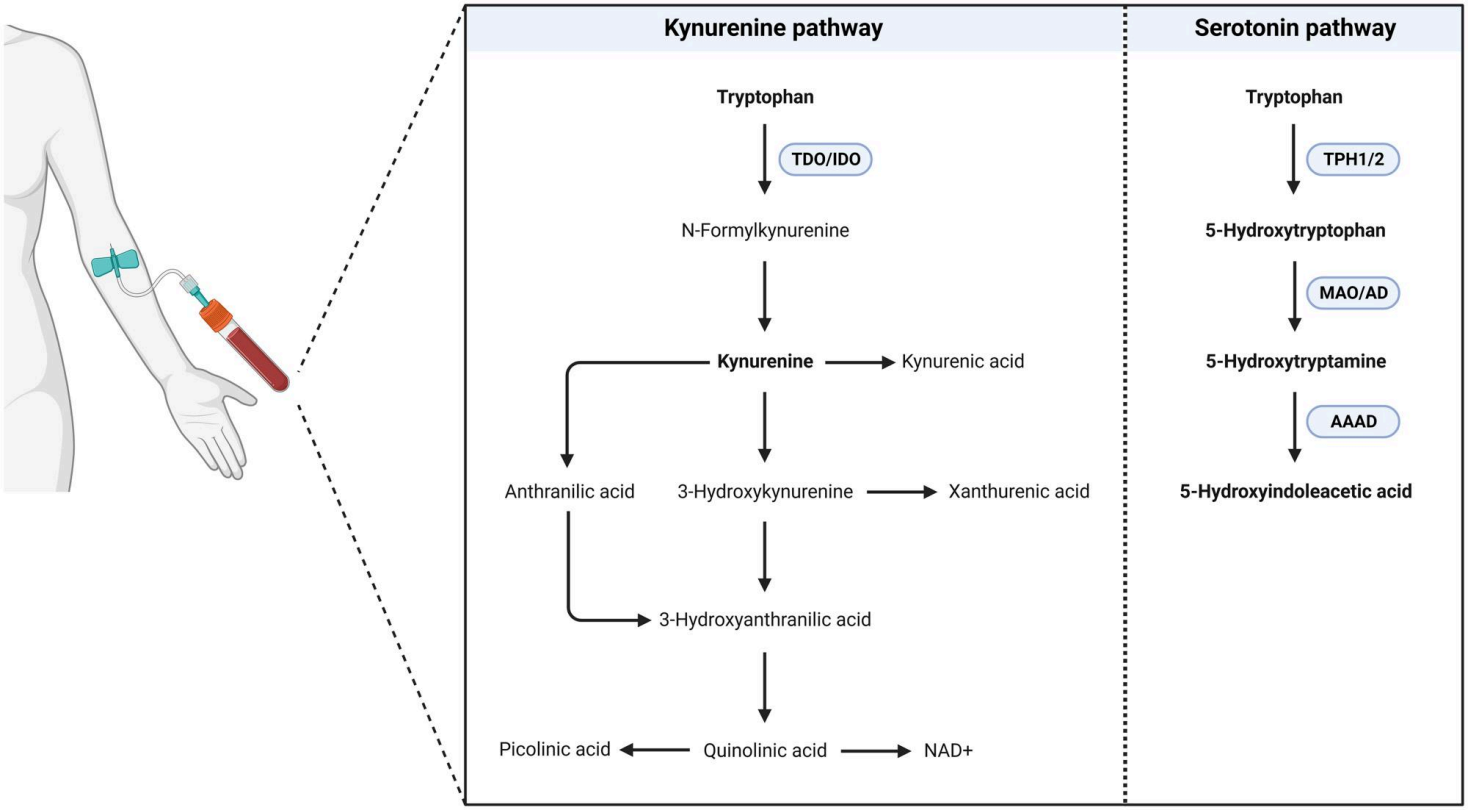
**Tryptophan metabolism along the kynurenine and serotonin pathway.** The analytes in bold were determined in maternal serum in the present study. AAAD: aromatic amino acid decarboxylase; AD: aldehyde dehydrogenase; IDO: indoleamine-2,3-dioxygenase; MAO: monoamine oxidase; TDO: tryptophan-2,3-dioxygenase; TPH: tryptophan hydroxylase.

The exact biological mechanisms underlying impaired embryonic growth and the subsequent adverse outcomes remain, however, largely unclear. It has been demonstrated that one-carbon metabolism is of key importance in the periconception period because of its role in the biosynthesis of DNA and proteins, and epigenetic modifications (Steegers-Theunissen *et al.*, 2013; Kalhan, 2016). Derangements in one-carbon metabolism as a result of poor maternal exposures have been associated with impaired embryonic and fetal growth, with implications for future health (Steegers-Theunissen, *et al.*, 2013). Another potential biological mechanism that contributes to fetal growth is the tryptophan (TRP) metabolism. TRP metabolism is linked to one-carbon metabolism as it provides methyl donors and shares substrates and cofactors (Steegers-Theunissen *et al.*, 2013). TRP is an essential amino acid, and its free concentration is dependent on protein and energy intake, and other exposures (Wurtman *et al.*, 2003; Badawy, 2017b). Physiologically, 95% of free TRP is metabolized through the kynurenine (KYN) pathway, with 1–5% through the serotonin pathway, and the rest of TRP is reserved for the indole pathway and protein synthesis (Fig. 1) (Bender, 1983; Oxenkrug, 2010). TRP metabolites are involved in processes essential to maintain pregnancy, including immune regulation, anti-oxidative processes, and regulation of vascular tone (Badawy, 2015; Broekhuizen *et al.*, 2021).

Throughout pregnancy, the TRP metabolite concentrations are closely regulated, and alterations of KYN pathway or serotonin pathway metabolites have been associated with pregnancy complications, such as pre-eclampsia and fetal growth restriction (FGR) (Gumusoglu *et al.*, 2021; van Zundert *et al.*, 2022a). In late pregnancy, maternal TRP and KYN concentrations were inversely associated with FGR (van Zundert, *et al.*, 2022a; Yaman *et al.*, 2023). Studies investigating TRP metabolism in the placenta showed that in placentas of FGR pregnancies the KYN pathway was downregulated, while the serotonin pathway was upregulated (Ranzil *et al.*, 2019). Animal studies also suggest that increased maternal serotonin pathway metabolite concentrations have a negative effect on fetal growth, illustrated by reduced fetal weight after administration of 5-hydroxytryptophan (5-HTP) and 5-hydroxytryptamine (5-HT) (Salas *et al.*, 2007).

To date, however, human studies investigating the role of the TRP metabolism in fetal growth are scarce and focus on late pregnancy or on placental TRP metabolism (van Zundert *et al.*, 2022a). To expand our understanding of the biological mechanisms involved in (patho)physiological embryonic and fetal growth, we studied the association between first trimester maternal TRP metabolites and embryonic growth. The secondary aim of this study was to investigate the associations between first trimester maternal TRP metabolites and fetal growth and birthweight, and the risk of small-for-gestational age (SGA) at birth.

## Materials and methods

### Study design and setting

This study was embedded within the Rotterdam Periconceptional Cohort (Predict Study), an ongoing prospective tertiary hospital-based cohort study with the aim to explore determinants of periconception parental health in relation to reproductive and pregnancy course and outcomes, and potential underlying mechanisms. The cohort profile has been described in detail previously (Steegers-Theunissen *et al.*, 2016; Rousian *et al.*, 2021). In short, women were eligible if they were older than 18 years, familiar with the Dutch language, and <11 weeks pregnant.

### Study population

**Figure 2.**
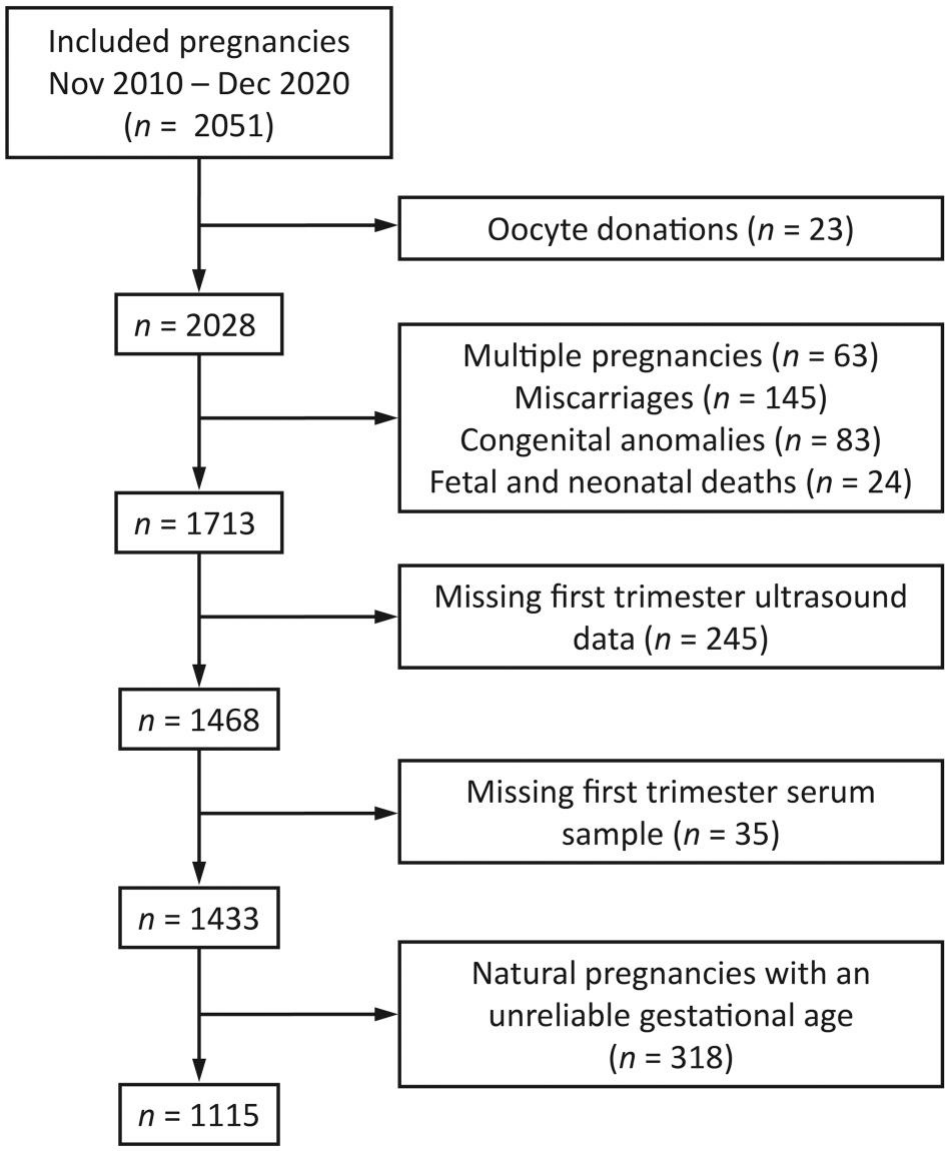
**Flowchart of the study population in an investigation of first trimester maternal tryptophan metabolism and embryonic and fetal growth**.

In total, 2051 pregnancies were included in the Predict Study between November 2010 and December 2020 (Fig. 2). We excluded pregnancies conceived after oocyte donation (n = 23), since no information was collected of the donors. In addition, pregnancies with an increased *a priori* risk of reduced embryonic growth were excluded: multiple pregnancies (n = 63), miscarriages (n = 145), congenital anomalies (n = 83), and fetal and neonatal deaths (n = 24). Then, pregnancies with missing first trimester 3D ultrasound imaging (n = 245) or missing first trimester maternal serum samples (n = 35) were excluded.

Since longitudinal crown–rump length (CRL) was one of the features of embryonic growth assessed in this study, and gestational age is the most important determinant of embryonic growth, gestational age was not based on the CRL. The gestational age was calculated using the first day of the last menstrual period (LMP) when women conceived naturally and had a regular menstrual cycle (25–31 days). The gestational age was adjusted for the duration of the menstrual cycle if the menstrual cycle deviated by 3–7 days from 28 days, with a fixed number of days each cycle.

The gestational age was determined using the CRL at 9 weeks of gestation; however, when women conceived naturally but had an irregular menstrual cycle (<21 days or >35 days), the LMP was missing or when the difference between the gestational age based on the LMP and the gestational age based on the CRL at 9 weeks of gestation was ≥7 days. For pregnancies conceived after IVF and ICSI, the gestational age was calculated from the conception date: the oocyte retrieval day minus 14 days for the fresh embryo transfers or minus 19 days for the cryopreserved embryo transfers (Steegers-Theunissen, *et al.*, 2016).

Pregnancies of which the gestational age was based on the CRL were excluded from the analysis with embryonic growth features as outcome (n = 318). As a result, the primary analysis was performed on a study sample consisting of 1115 pregnancies, and the secondary analysis was performed on a study sample of 1433 pregnancies.

### Data collection

#### TRP metabolites

Non-fasting venous blood was drawn during the hospital intake at 8.4 (interquartile range (IQR) = 7.3–9.4) weeks of gestation, and TRP metabolites were determined in the serum using a validated liquid chromatography (tandem) mass spectrometry (LC-MS/MS) method (van Zundert *et al.*, 2022b). The following TRP metabolites were determined: TRP, KYN, 5-HTP, 5-HT, and 5-hydroxyindole acetic acid (5-HIAA). The KYN/TRP and 5-HTP/TRP ratios were determined as they may provide an indication of the KYN pathway and serotonin pathway, respectively (Badawy and Guillemin, 2019).

#### Embryonic growth features

Embryonic growth features included CRL and embryonic volume (EV), which were measured using 3D ultrasound imaging and virtual reality techniques (Steegers-Theunissen *et al.*, 2016; Rousian *et al.*, 2021). These measurements have been proven reliable and reproducible (Rousian *et al.*, 2010). Transvaginal 3D ultrasounds were performed at ∼7, 9, and 11 weeks of gestation using a Voluson E8 or E10 (GE Healthcare, Austria) ultrasound machine. Accurate and precise 3D CRL and EV measurements were performed offline using 4D view software (GE Healthcare, Austria) and the V-Scope volume rendering application. A hologram of the embryo was created, which allowed for interaction and real depth perception. Manually, the signal of the uterus and umbilical cord was removed based on differences in echogenicity between the structures. The CRL was measured manually three times using a tracing tool, and the EV was measured using a semi-automated method (Steegers-Theunissen *et al.*, 2016; Rousian *et al.*, 2021).

#### Fetal growth parameters and birth outcomes

Fetal growth parameters were retrieved from the routine transabdominal mid-pregnancy anomaly scan at 20.1 (IQR = 19.9–20.6) weeks of gestation. Estimated fetal weight (EFW) was calculated using the Hadlock formula (Hadlock *et al.*, 1985):

$$\mathrm{EFW}=10^{\left(1.3596-0.00386\times AC\times FL+0.0064\times HC+0.00061\times \mathrm{BPD}\times AC+0.0424\times AC+0.174\times FL\right)}$$

EFW: estimated fetal weight; AC: abdominal circumference; FL: femur length; HC: head circumference; BPD: biparietal diameter.

Birthweight was retrieved from delivery reports. For both EFW and birthweight, standard scores (percentiles and *z*-scores) were calculated based on Dutch reference curves, adjusted for gestational age, and for gestational age and fetal sex, respectively (Gaillard *et al.*, 2011; Hoftiezer *et al.*, 2016). Percentiles were used to identify SGA cases, defined as a birthweight below the 10th percentile (Hoftiezer *et al.*, 2016).

#### Covariates

Data on maternal characteristics were collected via a general questionnaire and a validated food frequency questionnaire (FFQ), which were both filled out before the hospital intake during the first trimester of pregnancy (Feunekes *et al.*, 1993; Fayet *et al.*, 2011). The general questionnaire collected data on date of birth, geographical background, educational level, smoking, and folic acid supplement use. Based on the date of birth and the conception date, the age at conception was calculated. Geographical background was classified as either Western or non-Western, following the categorization proposed by Alders (2001). Educational levels were categorized into three groups based on the International Standard Classification of Education (ISCED): low (ISCED 0–2), intermediate (ISCED 3–4), and high (ISCED 5–8) (United Nations Educational Scientific and Cultural Organization, 2012). Smoking included any smoking during the periconception period. Folic acid supplement use was considered adequate in this study when initiated before conception. Information on maternal protein and energy intake were obtained from the FFQ taking into account the reliability using the Goldberg cut-off, which compared an individual’s reported energy intake to their basal metabolic rate and physical activity (Black, 2000). A comprehensive description of this method can be found in the recent publication of Smit *et al.* (2022). The completeness of the questionnaire was checked by a trained research nurse upon hospital intake. Additionally, anthropometric measurements were performed using a standard protocol and blood was drawn. BMI was calculated by dividing weight (kg) by the square of height (m). Data on conception mode, parity, and fetal sex were retrieved from medical records. Conception mode referred to the method through which pregnancy was achieved, encompassing naturally conceived pregnancies and IVF/ICSI pregnancies.

### Statistical analysis

Baseline characteristics, including periconception maternal characteristics, fetal and birth outcomes, and TRP metabolite concentrations, were presented as means with SD or as numbers of individuals. However, for 5-HIAA, owing to its right-skewed distribution, the median and IQR were used instead.

5-HIAA was (natural) log-transformed to obtain an approximate normal distribution for the analyses. In addition, a square root transformation for CRL and a cube root transformation for EV were adopted to obtain a constant variance and an approximate normal distribution of residuals provided the included covariates.

Multivariable mixed models were constructed to investigate the associations between maternal TRP metabolites and embryonic growth trajectories. Mixed models are flexible and can handle unbalanced data and meanwhile account for the correlated (repeated) measurements within each pregnancy by including a random intercept (Laird and Ware, 1982). Model 1 (crude model) was only adjusted for a cubic spline function of gestational age at the moment of the ultrasound. To identify potential confounders, a directed acyclic graph based on existing literature and a correlation matrix were constructed. Covariates additionally included in Model 2 (adjusted model) were gestational age at the blood draw, maternal age, geographical background, educational level, smoking, folic acid supplement use, protein intake/energy intake, BMI, conception mode, parity, and fetal sex. Including the covariates as nonlinear terms and including all possible two-way interactions did not improve the fit based on the Akaike information criterion and Bayesian information criterion.

Multivariable linear regression models were used to assess the associations between TRP metabolites and EFW and birthweight. Since the EFW *z*-scores were already adjusted for gestational age and SGA for gestational age and fetal sex, these covariates were not included in the multivariable model. Model 2 included the covariates gestational age at the blood draw, maternal age, geographical background, educational level, smoking, folic acid supplement use, protein intake/energy intake, BMI, conception mode, parity, and fetal sex (only for EFW). Finally, multivariable logistic regression models, using the same covariates as in the linear regression model for birthweight, were built to investigate the associations between TRP metabolites and SGA.

Results were displayed as effect estimates (β or odds ratio (OR)) with 95% CI. All statistical analyses were performed using R version 4.2.1 and IBM SPSS Statistics for Windows version 28 (IBM Corp, 2021; R Core Team, 2022).

### Ethical approval

Ethical clearance was sought and obtained from the local Medical Ethical Committee of the Erasmus MC, University Medical Center, Rotterdam and the Central Committee on Research The Hague (15 October 2004, MEC-2004-277; Steegers-Theunissen, *et al.*, 2016). All participants gave written informed consent at enrolment.

## Results

### Baseline characteristics

Table 1 shows the baseline characteristics of the total study population (n = 1115), including information on the periconception maternal characteristics, fetal and birth outcomes, and absolute maternal TRP concentrations. In Supplementary Table S1, the baseline characteristics of the study sample used for the secondary analysis (n = 1433) are presented, which were comparable with those described below. Supplementary Table S2 shows the baseline characteristics of the total excluded population and of those excluded based on missing first trimester 3D ultrasound data or missing first trimester serum samples. Those who were excluded from the analysis tended to have higher BMI and lower educational level, and were more likely to smoke, conceive naturally, be multiparous, and have an inadequate folic acid supplement intake.

**Table 1.** Periconception maternal characteristics, fetal and birth outcomes, and tryptophan metabolite concentrations of the total study population.

Baseline characteristics	Total study population (*n *=* *1115)
**Periconception maternal characteristics**	

Age at conception (years)	32.5 (4.5)
*Missing*	*0*
BMI (kg/m^2^)	25.5 (4.8)
*Missing*	*30*
Geographical background	
Western	918 (86.3)
Non-Western	146 (13.7)
*Missing*	*51*
Educational level	
Low	73 (6.9)
Middle	366 (34.4)
High	626 (58.8)
*Missing*	*50*
Parity	
Nulliparous	597 (54.3)
Multiparous	503 (45.7)
*Missing*	*15*
Conception mode	
Natural	567 (50.9)
IVF/ICSI	548 (49.1)
*Missing*	*0*
Any smoking	
Yes	147 (13.8)
No	917 (86.2)
*Missing*	*51*
Any alcohol use	
Yes	308 (29.0)
No	755 (71.0)
*Missing*	*52*
Any drug use	
Yes	15 (1.4)
No	1049 (98.6)
*Missing*	*51*
Folic acid supplement use^a^	
Adequate	896 (84.4)
Inadequate	166 (15.6)
*Missing*	*53*
Energy intake (kJ/day)	8215.0 (2223.2)
*Missing*	*108*
*Unreliable*	*216*
Protein intake/energy intake (grams/day)	72.6 (19.6)
*Missing*	*108*
*Unreliable*	*216*

**Fetal and birth outcomes**	

Fetal sex	
Girl	530 (50.6)
Boy	518 (49.4)
*Missing*	*67*
EFW mid-pregnancy (g)	352.6 (46.9)
*Missing*	*102*
EFW mid-pregnancy (*z*-score)	0.5 (1.0)
*Missing*	*105*
Gestational age at delivery (weeks)	38.8 (2.0)
*Missing*	*69*
Birthweight (grams)	3280.5 (580.4)
*Missing*	*75*
Birthweight (*z*-score)	–0.10 (1.1)
*Missing*	*80*
SGA (birthweight <p10)	
Yes	129 (11.6)
No	906 (81.3)
*Missing*	*80*

**Tryptophan metabolites**	

TRP (µmol/l)	56.3 (9.5)
	28.8–101.5
KYN (µmol/l)	1.5 (0.3)
	0.7–3.5
5-HTP (nmol/l)	4.5 (1.7)
	1.1–28.9
5-HT (nmol/l)	676.0 (284.9)
	16.1–2180.2
5-HIAA (nmol/l)	38.8 (31.8–51.3)
	15.9 508.2
Natural log of 5-HIAA (nmol/l)	3.8 (0.5)
	2.8–6.2

a Folic acid supplement use was considered adequate when initiated before conception. Continuous data are presented as mean with SD or median with interquartile range (5-HIAA), and categorical data as numbers with percentages. For the tryptophan metabolites also the range (minimum–maximum) is given.

5-HIAA: 5-hydroxyindoleacetic acid; 5-HT: 5-hydroxytryptamine; 5-HTP: 5-hydroxytryptophan; AC: abdominal circumference; EFW: estimated fetal weight; KYN: kynurenine; TRP: tryptophan.

#### Periconception maternal characteristics

The mean maternal age at conception was 32.5 (SD = 4.5) years, and the mean BMI was 25.5 (SD = 4.8) kg/m^2^ (Table 1). Women mostly had a Western geographical background (n = 918 [86.3%]), were highly educated (n = 626 [58.8%]), and often nulliparous (n = 597 [54.3%]). Slightly less than half of the women conceived after IVF/ICSI treatment (49.1%) and the others conceived naturally (50.9%). During the periconception period, one-third of the women consumed alcohol (n = 308 [29.0%]), 147 (13.8%) women smoked, and a few women (n = 15 [1.4%]) used drugs. Most women initiated folic acid supplement use before conception (n = 896 [84.4%]), which was considered adequate. Women consumed daily 72.6 g protein (SD = 19.6) and 8215.0 kJ (SD = 2223.2) energy, both of which are in accordance with current recommendations in obstetric practice (Most *et al.*, 2019; Murphy *et al.*, 2021).

#### Fetal and birth outcomes

Mid-pregnancy, at 20.1 (IQR = 19.9–20.6) weeks of gestation, the EFW in our study population was slightly higher than in the general Dutch population (*z*-score = 0.5 (SD = 1.0)), while the birthweight was slightly lower than in the general Dutch population (*z*-score = –0.10 (SD = 1.1)). Women delivered at a mean gestational age of 38.8 (SD = 2.0) weeks, and in 11.6% (n = 129), this concerned a SGA-baby.

### Embryonic growth

**Figure 3.**
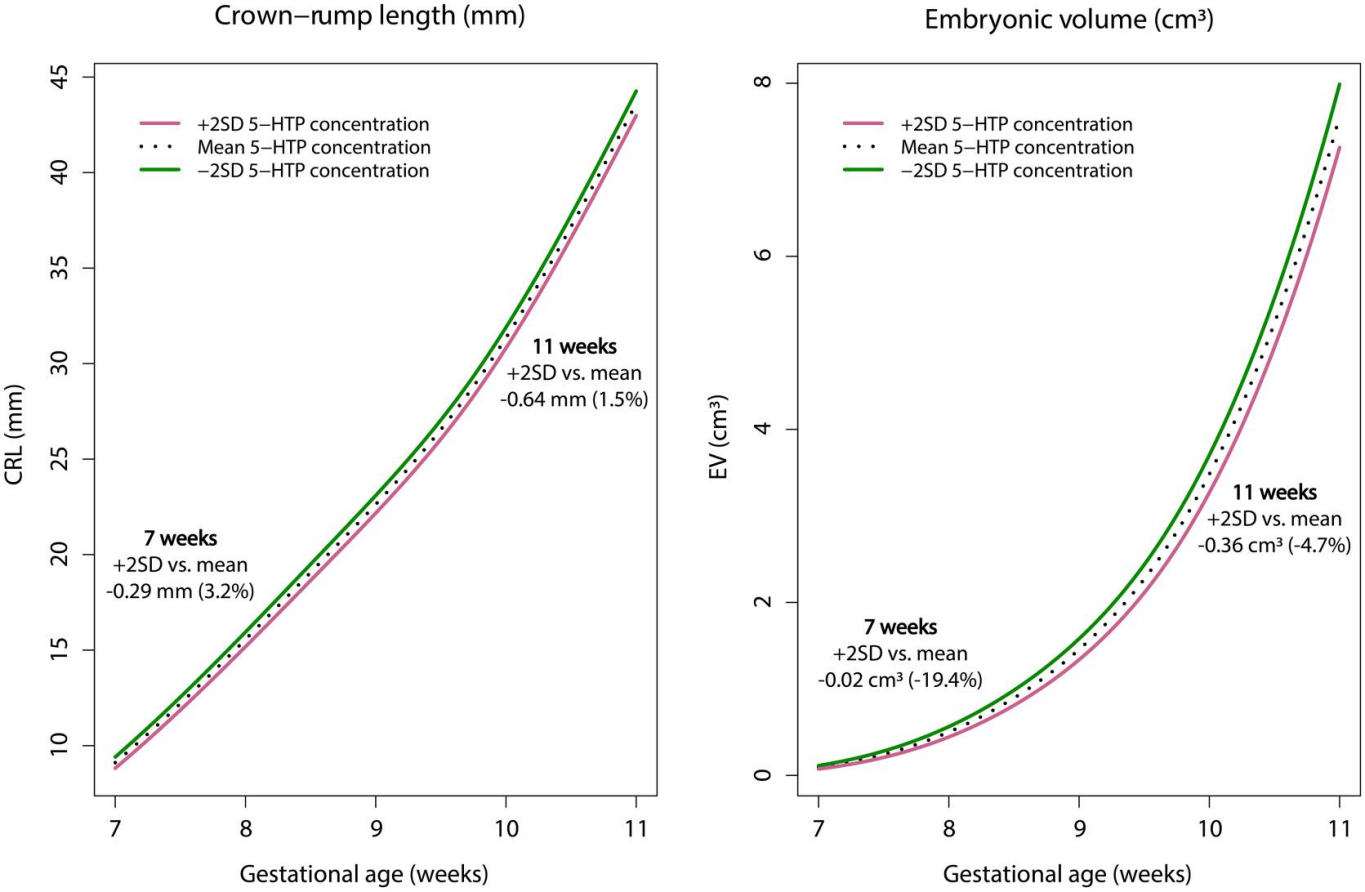
**Median crown–rump length and embryonic volume trajectories during the first trimester of pregnancy stratified for 5-hydroxytryptophan concentration.** The trajectories were adjusted for gestational age, maternal age, geographical background, educational level, smoking, folic acid supplement use, protein intake/energy intake, BMI, conception mode, parity, and fetal sex. 5-HTP: 5-hydroxytryptophan; CRL: crown–rump length; EV: embryonic volume.

The associations between maternal TRP metabolite concentrations and CRL and EV trajectories are presented in Table 2. Results described below are based on the adjusted model, unless stated otherwise. An inverse association was found between maternal 5-HTP concentrations and both CRL and EV trajectories (√CRL: β = –0.015, 95% CI = –0.028 to –0.001; ^3^√EV: β = –0.009, 95% CI = –0.016 to –0.003). The median decrease in CRL and EV during the first trimester of pregnancy for +2SD 5-HTP concentrations compared to mean 5-HTP concentrations is illustrated in Fig. 3. In addition, the maternal 5-HTP/TRP ratio was inversely associated with both CRL and EV trajectories (√CRL: β = –0.843, 95% CI = –1.597 to –0.088; ^3^√EV: β = –0.529, 95% CI = –0.902 to –0.157). No associations were found between other TRP metabolites and CRL or EV trajectories.

**Table 2.** Associations between first trimester maternal tryptophan metabolites and embryonic growth (crown–rump length and embryonic volume trajectories) during the first trimester of pregnancy in the total study population.

Study sample primary analysis (*n *=* *1115)		√CRL (mm)			^3^√EV (cm^3^)		
		β	95% CI		β	95% CI	
**TRP (µmol/l)**	Model 1	0.000	–0.002	0.002	0.000	–0.001	0.001
	Model 2	0.000	–0.002	0.002	–0.000	–0.001	0.001
**KYN (µmol/l)**	Model 1	–0.008	–0.062	0.046	–0.004	–0.031	0.023
	Model 2	–0.007	–0.078	0.063	0.002	–0.033	0.038
**5-HTP (nmol/l)**	Model 1	0.001	–0.009	0.011	–0.002	–0.007	0.003
	Model 2	–0.015*	–0.028	–0.001	–0.009*	–0.016	–0.003
**5-HT (nmol/l)**	Model 1	0.000	0.000	0.000	0.000	0.000	0.000
	Model 2	–0.000	–0.000	0.000	–0.000	–0.000	0.000
**Ln 5-HIAA (nmol/l)**	Model 1	0.019	–0.016	0.054	0.017	–0.001	0.035
	Model 2	0.001	–0.043	0.045	0.007	–0.015	0.029
**KYN/TRP ratio**	Model 1	–0.257	–2.955	2.440	–0.030	–1.396	1.337
	Model 2	–0.264	–3.783	3.255	0.325	–1.425	2.075
**5-HTP/TRP ratio · 10^3^**	Model 1	–0.035	–0.606	0.537	–0.148	–0.436	0.140
	Model 2	–0.843*	–1.597	–0.088	–0.529*	–0.902	–0.157

Mixed models are used. Model 1 is adjusted for gestational age at the ultrasound. Model 2 is additionally adjusted for gestational age at the blood draw, maternal age, geographical origin, educational level, smoking, folic acid supplement use, protein intake/energy intake, BMI, conception mode, parity, and fetal sex. A square root transformation for CRL and a cube root transformation for EV were adopted to obtain a constant variance and an approximate normal distribution of residuals provided the included covariates.

* *P*-value ≤ 0.05 (statistically significant).

5-HIAA: 5-hydroxyindoleacetic acid; 5-HT: 5-hydroxytryptamine; 5-HTP/TRP ratio: 5-hydroxytryptophan/tryptophan ratio; 5-HTP: 5-hydroxytryptophan; CRL: crown–rump length; EV: embryonic volume; KYN/TRP ratio: kynurenine/tryptophan ratio; KYN: kynurenine; TRP: tryptophan.

### Fetal growth and birth outcomes

Tables 3 and 4 show the associations between maternal TRP metabolite concentrations and EFW and SGA, respectively. In Supplementary Table S3, the associations between maternal TRP metabolite concentrations and birthweight are presented. Inverse associations were found between maternal 5-HTP concentrations and both EFW and birthweight, though not statistically significant. Comparable results were found for the maternal 5-HTP/TRP ratio. In addition, a higher maternal 5-HTP/TRP ratio was associated with an increased risk of SGA (OR = 1.006, 95% CI = 1.000–1.013). In contrast, increased maternal KYN concentrations were associated with higher birthweight and a reduced risk of SGA in the unadjusted models (birthweight *z*-score: β = 0.188, 95% CI = 0.010–0.365, SGA: OR = 1.006, 95% CI = 1.000–1.013). The analysis revealed no other associations between TRP metabolites and fetal growth or birth outcomes.

**Table 3.** Associations between first trimester maternal tryptophan metabolites and standardized estimated fetal weight (*z*-scores) in the total study population.

Study sample secondary analysis (n = 1433)		EFW (*z*-score)		
		β	95% CI	
**TRP (µmol/l)**	Model 1	0.004	–0.002	0.010
	Model 2	0.001	–0.006	0.008
**KYN (µmol/l)**	Model 1	0.045	–0.131	0.221
	Model 2	–0.057	–0.275	0.160
**5-HTP (nmol/l)**	Model 1	0.023	–0.011	0.057
	Model 2	–0.030	–0.073	0.012
**5-HT (nmol/l)**	Model 1	0.000	–0.001	0.000
	Model 2	0.000	–0.000	0.000
**Ln 5-HIAA (nmol/l)**	Model 1	0.063	–0.056	0.182
	Model 2	–0.067	–0.206	0.072
**KYN/TRP ratio · 10^3^**	Model 1	–0.002	–0.011	0.006
	Model 2	–0.004	–0.014	0.007
**5-HTP/TRP ratio · 10^6^**	Model 1	0.000	–0.002	0.002
	Model 2	–0.002	–0.004	0.000

Linear regression models are used. Model 1 is the crude model. Model 2 is adjusted for gestational age at the blood draw, maternal age, geographical origin, educational level, smoking, folic acid supplement use, protein intake/energy intake, BMI, conception mode, parity, and fetal sex.

* *P*-value ≤ 0.05 (statistically significant).

5-HIAA: 5-hydroxyindoleacetic acid; 5-HT: 5-hydroxytryptamine; 5-HTTP: 5-hydroxytryptophan; EFW: standardized estimated fetal weight; KYN: kynurenine; TRP: tryptophan.

**Table 4.** Associations between first trimester maternal tryptophan metabolites and the risk of small-for-gestational age at birth in the total study population.

Study sample secondary analysis (*n *=* *1433)		Small-for-gestational age		
		Odds ratio	95% CI	
**TRP (µmol/l)**	Model 1	0.994	0.978	1.011
	Model 2	0.995	0.973	1.016
**KYN (µmol/l)**	Model 1	0.548*	0.320	0.921
	Model 2	0.679	0.330	1.338
**5-HTP (nmol/l)**	Model 1	1.035	0.943	1.125
	Model 2	1.101	0.978	1.240
**5-HT (nmol/l)**	Model 1	1.000	1.000	1.001
	Model 2	1.000	0.999	1.001
**Ln 5-HIAA (nmol/l)**	Model 1	0.912	0.645	1.259
	Model 2	1.000	0.646	1.498
**KYN/TRP ratio · 10^3^**	Model 1	0.987	0.962	1.012
	Model 2	1.001	0.967	1.035
**5-HTP/TRP ratio · 10^6^**	Model 1	1.003	0.998	1.008
	Model 2	1.006*	1.000	1.013

Small-for-gestational age at birth was defined as a birthweight below the 10^th^ percentile. Logistic regression models are used. Model 1 is the crude model. Model 2 is adjusted for gestational age at the blood draw, maternal age, geographical origin, educational level, smoking, folic acid supplement use, protein intake/energy intake, BMI, conception mode, and parity.

* *P*-value ≤ 0.05 (statistically significant).

5-HIAA, 5-hydroxyindoleacetic acid; 5-HT: 5-hydroxytryptamine; 5-HTTP: 5-hydroxytryptophan; KYN: kynurenine; TRP: tryptophan.

## Discussion

### Summary of findings

Table 5 summarizes the associations found in this study between first trimester maternal TRP metabolites and embryonic growth, fetal growth, and birth outcomes. It shows that increased first trimester maternal 5-HTP concentrations are associated with a smaller embryo, expressed by reduced CRL and EV trajectories. In addition, our findings suggest that higher first trimester maternal 5-HTP concentrations are also associated with lower EFW and birthweight, and with an increased risk of SGA, while higher maternal KYN concentrations are associated with a higher birthweight, and a reduced risk of SGA.

**Table 5.** Summary of associations between first trimester maternal tryptophan metabolites and embryonic growth, fetal growth, and birth outcomes in the total study population.

	CRL	EV	EFW	BW	SGA-risk
**TRP**	●	●	●	**●**	**●**
**KYN**	●	●	●	↑	↓
**5-HTP**	↓*	↓*	↓	↓	↑
**5-HT**	●	●	●	●	●
**Ln 5-HIAA**	●	●	●	●	●
**KYN/TRP**	●	●	●	●	●
**5-HTP/TRP**	↓*	↓*	↓	↓	↑*

↑: positive association; ↓: negative association; ●: no association.

* *P*-value ≤ 0.05 (statistically significant in the adjusted model).

5-HIAA: 5-hydroxyindoleacetic acid; 5-HT: 5-hydroxytryptamine; 5-HTP: 5-hydroxytryptophan; BW: birthweight; CRL: crown–rump length; EFW: estimated fetal weight; EV: embryonic volume; KYN: kynurenine; SGA: small-for-gestational age; TRP: tryptophan.

### Comparison with existing literature and interpretation

Studies investigating maternal TRP metabolites in relation to fetal growth or birth outcomes are scarce (van Zundert *et al.*, 2022a). In fact, this is the first human study that found an inverse association between first trimester maternal 5-HTP concentrations and embryonic and fetal growth. The effect estimates of the association between first trimester maternal 5-HTP and embryonic growth (√CRL: β = –0.015, 95% CI = –0.028 to –0.001; ^3^√EV: β = –0.009, 95% CI = –0.016 to –0.003) were smaller than those of the association between periconception smoking and embryonic growth (√CRL: β = –0.055, 95% CI = –0.159 to –0.006; ^3^√EV: β = –0.042, 95% CI = –0.089 to 0.006), but larger than those of the association between periconception alcohol use and embryonic growth (√CRL: β = –0.003, 95% CI = –0.005 to –0.003; ^3^√EV: β = –0.005, 95% CI = –0.040 to 0.030) (van Uitert *et al.*, 2013a; van Dijk *et al.*, 2018).

There are several possible explanations for this finding. Insights from animal studies suggest that maternal 5-HTP can directly inhibit fetal growth by passing into the fetal circulation. These animal studies showed that 5-HTP can induce developmental arrest, inhibit growth, and increase cell death (Gordeeva and Gordeev, 2021; Gordeeva and Safandeev, 2021). In humans, there is increasing evidence that initially nutrition of the embryo is histiotrophic, with secretion of nutrients by uterine glands into the intervillous space, which are taken up by the trophoblast (Burton *et al.*, 2001). The nutrients diffuse into the coelomic fluid from which they may be absorbed by the (secondary) yolk sac, and then reach the embryo. There is a transition toward hemotrophic nutrition when the maternal placental circulation is established (Burton *et al.*, 2001). Additional research is needed to determine whether maternal 5-HTP indeed reaches the embryo through histiotrophic nutrition.

Another explanation for the inverse association between maternal 5-HTP concentrations and fetal growth may be the involvement of 5-HTP in placental development. Support for this biological mechanism can be found in an animal study that reported reduced fetal and placental weights after treating pregnant rats with 5-HTP (Salas *et al.*, 2007). This study postulated that treatment with 5-HTP increases placental vascular resistance. Consequently, reduced utero-placental blood flow can potentially affect fetal growth negatively.

Disruption of the one-carbon metabolism could also underlie the inverse association between first trimester maternal 5-HTP and embryonic and fetal growth. TRP metabolism provides one-carbon units for the folate cycle and shares cofactors with one-carbon metabolism, which is crucial for epigenetic programming of cells and tissues (Steegers-Theunissen *et al.*, 2013; Shibata *et al.*, 2015). A deficiency of a shared cofactor, for example vitamin B6, can lead to increased 5-HTP and homocysteine concentrations (Shabbir *et al.*, 2013; Steegers-Theunissen *et al.*, 2013). Increased homocysteine is a marker of derangements in one-carbon metabolism, which has been associated with impaired embryonic and placental development (Parisi *et al.*, 2017; Hoek *et al.*, 2021; Rubini *et al.*, 2021, 2022).

No associations between 5-HT and embryonic and fetal growth were found in the present study. This can be explained by the methodological difficulty to accurately measure serum 5-HT owing to leakage of 5-HT from thrombocytes during the clotting process. Nevertheless, the concentrations found in our study are comparable with those reported in earlier studies (Ernberg *et al.*, 2000; Comai *et al.*, 2010; Lindström *et al.*, 2018; Zhao *et al.*, 2022). Future research should consider assessing platelet count and 5-HT in other blood fractions as well, such as in platelet-rich plasma (Korse *et al.*, 2017).

Another interesting finding was the association between higher maternal KYN concentrations during the first trimester of pregnancy and a reduced risk of SGA. These associations were only statistically significant in the unadjusted model, and therefore need to be interpreted with caution. Although reference values for maternal KYN concentrations during the first trimester of pregnancy are currently lacking, the concentrations observed in our study align with those recently reported (Sha *et al.*, 2022). Even though maternal KYN concentrations remain relatively stable throughout pregnancy, we accounted for timing of the blood draw in the first trimester of pregnancy by adjusting for gestational age at the moment of the blood draw, which occurred at hospital intake between 7 and 9 weeks of gestation (Schröcksnadel *et al.*, 1996; van Zundert, *et al.*, 2022a). However, as expected, the timing of the blood draw did not affect the results substantially.

In line with our findings, a recent study reported lower maternal KYN concentrations in the third trimester in pregnancies complicated by FGR (Yaman *et al.*, 2023). Explanations for the positive association between first trimester maternal KYN concentrations and SGA involve the several essential functions of the KYN pathway during pregnancy, including its role in neovascularization, vasodilatation of placental arteries, and immune regulation (Wang *et al.*, 2010; Mondal *et al.*, 2016; Badawy, 2017a; Broekhuizen, *et al.*, 2021; Worton *et al.*, 2021). These processes are all important for embryonic and fetal growth, and placental development and functioning. No clear positive associations were found with fetal growth. Since the assessment of fetal growth was based on EFW calculated from the mid-pregnancy anomaly scan, measurements errors cannot be ruled out, which may have weakened the observed effects (Milner and Arezina, 2018).

First trimester maternal KYN concentrations were also not positively associated with embryonic growth in this study. Excess KYN concentrations can induce oxidative stress and dysregulate apoptosis, which may affect embryonic growth negatively (Forrest *et al.*, 2004; Wang *et al.*, 2014). Possibly, the potential harmful effects of increased KYN concentrations may outweigh the relaxing effects on placental arteries, since the embryo is dependent on histiotrophic nutrition rather than on hemotrophic nutrition via the placenta during early pregnancy (Burton, *et al.*, 2001).

### Strengths and limitations

To the best of our knowledge, this is the first human study that investigated associations between maternal TRP metabolites in the first trimester of pregnancy and embryonic growth. The primary strengths of our study lie in its periconceptional prospective design, enabling comprehensive data collection on various periconception maternal characteristics through validated general and food questionnaires (Feunekes *et al.*, 1993; Fayet *et al.*, 2011). This allowed building extensive multivariable models to adjust for potential confounders including dietary factors, such as protein and energy intake, although the presence of residual confounding from unobserved and unknown factors, such as comorbidities and medication use (e.g. selective serotonin reuptake inhibitors and interferon alpha), cannot be ruled out (Correia and Vale, 2022). In addition, standardized longitudinal embryonic growth measurements were conducted using 3D ultrasound scans and virtual reality systems, which have been proven highly accurate and reliable (Rousian, *et al.*, 2010, 2021). Furthermore, the TRP concentrations were determined using a recently published LC-MS/MS method, which has been validated in women in early pregnancy, allowing for sensitive and accurate measurements (van Zundert *et al.*, 2022b). Possible selection bias exists if associations differ between excluded and included participants; however, there is no reason to assume, for example, that lower first trimester maternal 5-HTP concentrations result in reduced embryonic growth in the excluded population compared to the study population, especially since participants were unaware of the study’s associations, and the outcome did not impact their pregnancy course or health.

Total TRP was measured instead of free TRP, as free TRP can be influenced by various factors that were not optimal in this study, such as the absence of fasting blood samples (Badawy, 2010). However, this limits the interpretation of maternal TRP concentrations, as only free TRP is available for protein synthesis and metabolic processes. Another potential limitation is the recruitment of women from a tertiary hospital, which may limit external validity. We expect that the direction of the associations will remain consistent in the general population, and there is a possibility that they might even be stronger. Considering the explorative character of this study, we accepted a higher type I error rate, and have not corrected for multiple testing using statistical procedures.

### Implications and future research

Our findings provide valuable insights into the role of the maternal TRP metabolism during the first trimester of pregnancy in the physiology of embryonic and fetal growth. Given the pivotal role of embryonic and fetal growth in determining birth outcomes, and long-term health, including potential transgenerational effects, understanding the associated metabolic processes is crucial to achieve the ultimate goal of optimizing embryonic and fetal growth and reducing the occurrence of adverse birth outcomes (Steegers-Theunissen *et al.*, 2013). Our findings suggest that alterations in maternal TRP metabolism, particularly leading to increased 5-HTP concentrations, may contribute to impaired embryonic and fetal growth. This novel finding contributes to our comprehension of the pathophysiology of impaired embryonic and fetal growth, and provides potential preventative and therapeutic targets that warrant further investigation to assess their value in periconception care. A well-balanced diet containing an appropriate intake of macro- and micronutrients is recommended, and vigilance is required when making future dietary recommendations for TRP intake. Future research is needed to identify modifiable factors that influence maternal TRP metabolism, which can be addressed in periconception care to optimize maternal TRP metabolism, and subsequently enhance embryonic and fetal growth. Despite the promising results, additional research within large prospective cohorts and intervention studies is required to validate our findings and to further elucidate the mechanisms through which alterations of maternal TRP metabolites affect embryonic and fetal growth. To develop a full picture of the maternal TRP metabolism, free TRP also needs to be determined in future studies. To measure free TRP accurately, fasting blood samples collected and stored under strictly controlled conditions are required (Badawy, 2010). This remains a challenging task for future studies.

## Conclusion

In conclusion, alterations of the maternal TRP metabolism during the first trimester of pregnancy, specifically increased 5-HTP concentrations, are associated with a smaller embryo, and potentially with a smaller fetus and a higher risk of SGA. In contrast, increased maternal KYN concentrations during the first trimester of pregnancy may be associated with a larger fetus and a lower risk of SGA. The findings of this study indicate that maternal TRP metabolism during the first trimester of pregnancy potentially plays a role in the (patho)physiology of embryonic and fetal growth.

## Supplementary Material

deae046_Supplementary_Table_S1

deae046_Supplementary_Table_S2

deae046_Supplementary_Table_S3

## Data Availability

The data underlying this article will be shared upon reasonable request to the corresponding author.

## References

[deae046-B1] Abdollahi S , Soltani S, de Souza RJ, Forbes SC, Toupchian O, Salehi-Abargouei A. Associations between maternal dietary patterns and perinatal outcomes: a systematic review and meta-analysis of cohort studies. Adv Nutr 2021;12:1332–1352. 33508080 10.1093/advances/nmaa156 PMC8321866

[deae046-B2] Alders M. Classification of the population with a foreign background in the Netherlands. In: *Paper for the conference “The measure and mismeasure of populations. The statistical use of ethnic and racial categories in multicultural societies”.* 2001. Paris: Citeseer, p. 18. Statistics Netherlands.

[deae046-B3] Badawy AA. Plasma free tryptophan revisited: what you need to know and do before measuring it. J Psychopharmacol 2010;24:809–815. 19074538 10.1177/0269881108098965

[deae046-B4] Badawy AA. Tryptophan metabolism, disposition and utilization in pregnancy. Biosci Rep 2015;35:1–16. 10.1042/BSR20150197 PMC4626867 26381576

[deae046-B5] Badawy AA. Kynurenine pathway of tryptophan metabolism: regulatory and functional aspects. Int J Tryptophan Res2017a;10:1178646917691938. 28469468 10.1177/1178646917691938 PMC5398323

[deae046-B6] Badawy AA. Tryptophan availability for kynurenine pathway metabolism across the life span: Control mechanisms and focus on aging, exercise, diet and nutritional supplements. Neuropharmacology2017b;112:248–263. 26617070 10.1016/j.neuropharm.2015.11.015

[deae046-B7] Badawy AA , Guillemin G. The plasma [kynurenine]/[tryptophan] ratio and indoleamine 2,3-dioxygenase: time for appraisal. Int J Tryptophan Res 2019;12:1178646919868978. 31488951 10.1177/1178646919868978 PMC6710706

[deae046-B8] Bender DA. Biochemistry of tryptophan in health and disease. Mol Aspects Med 1983;6:101–197. 6371429 10.1016/0098-2997(83)90005-5

[deae046-B9] Black AE. Critical evaluation of energy intake using the Goldberg cut-off for energy intake: basal metabolic rate. A practical guide to its calculation, use and limitations. Int J Obes Relat Metab Disord 2000;24:1119–1130. 11033980 10.1038/sj.ijo.0801376

[deae046-B10] Broekhuizen M , Danser AHJ, Reiss IKM, Merkus D. The function of the kynurenine pathway in the placenta: a novel pharmacotherapeutic target? Int J Environ Res Public Health 2021;18:11545. 34770059 10.3390/ijerph182111545 PMC8582682

[deae046-B11] Burton GJ , Hempstock J, Jauniaux E. Nutrition of the human fetus during the first trimester—a review. Placenta 2001;22 Suppl A:S70–S77. 11312634 10.1053/plac.2001.0639

[deae046-B12] Comai S , Bertazzo A, Carretti N, Podfigurna-Stopa A, Luisi S, Costa CV. Serum levels of tryptophan, 5-hydroxytryptophan and serotonin in patients affected with different forms of amenorrhea. Int J Tryptophan Res 2010;3:69–75. 22084589 10.4137/ijtr.s3804 PMC3195241

[deae046-B13] Correia AS , Vale N. Tryptophan metabolism in depression: a narrative review with a focus on serotonin and kynurenine pathways. Int J Mol Sci 2022;23:8493. 35955633 10.3390/ijms23158493 PMC9369076

[deae046-B14] Dickey RP , Gasser RF. Ultrasound evidence for variability in the size and development of normal human embryos before the tenth post-insemination week after assisted reproductive technologies. Hum Reprod 1993;8:331–337. 8473443 10.1093/oxfordjournals.humrep.a138046

[deae046-B15] Ernberg M , Voog U, Alstergren P, Lundeberg T, Kopp S. Plasma and serum serotonin levels and their relationship to orofacial pain and anxiety in fibromyalgia. J Orofac Pain 2000;14:37–46. 11203736

[deae046-B16] Fayet F , Flood V, Petocz P, Samman S. Relative and biomarker-based validity of a food frequency questionnaire that measures the intakes of vitamin B(12), folate, iron, and zinc in young women. Nutr Res 2011;31:14–20. 21310301 10.1016/j.nutres.2010.12.004

[deae046-B17] Feunekes GI , Van Staveren WA, De Vries JH, Burema J, Hautvast JG. Relative and biomarker-based validity of a food-frequency questionnaire estimating intake of fats and cholesterol. Am J Clin Nutr 1993;58:489–496. 8379504 10.1093/ajcn/58.4.489

[deae046-B18] Forrest CM , Mackay GM, Stoy N, Egerton M, Christofides J, Stone TW, Darlington LG. Tryptophan loading induces oxidative stress. Free Radic Res 2004;38:1167–1171. 15621693 10.1080/10715760400011437

[deae046-B19] Gaillard R , de Ridder MA, Verburg BO, Witteman JC, Mackenbach JP, Moll HA, Hofman A, Steegers EA, Jaddoe VW. Individually customised fetal weight charts derived from ultrasound measurements: the Generation R Study. Eur J Epidemiol 2011;26:919–926. 22083366 10.1007/s10654-011-9629-7 PMC3253277

[deae046-B20] Gluckman PD , Hanson MA, Buklijas T. A conceptual framework for the developmental origins of health and disease. J Dev Orig Health Dis 2010;1:6–18. 25142928 10.1017/S2040174409990171

[deae046-B21] Gordeeva O , Gordeev A. Comparative assessment of toxic responses in 3D embryoid body differentiation model and mouse early embryos treated with 5-hydroxytryptophan. Arch Toxicol 2021;95:253–269. 32926198 10.1007/s00204-020-02909-w

[deae046-B22] Gordeeva O , Safandeev V. 5-Hydroxytryptophan (5-HTP)-induced intracellular syndrome in mouse non-neural embryonic cells is associated with inhibited proliferation and cell death. Neuropharmacology 2021;195:107862. 31778690 10.1016/j.neuropharm.2019.107862

[deae046-B23] Gumusoglu S , Scroggins S, Vignato J, Santillan D, Santillan M. The serotonin-immune axis in preeclampsia. Curr Hypertens Rep 2021;23:37. 34351543 10.1007/s11906-021-01155-4 PMC8435353

[deae046-B24] Hadlock FP , Harrist RB, Sharman RS, Deter RL, Park SK. Estimation of fetal weight with the use of head, body, and femur measurements—a prospective study. Am J Obstet Gynecol 1985;151:333–337. 3881966 10.1016/0002-9378(85)90298-4

[deae046-B25] Hoek J , Schoenmakers S, Ringelberg B, Reijnders IF, Willemsen SP, De Rijke YB, Mulders A, Steegers-Theunissen RPM. Periconceptional maternal and paternal homocysteine levels and early utero-placental (vascular) growth trajectories: The Rotterdam periconception cohort. Placenta 2021;115:45–52. 34560327 10.1016/j.placenta.2021.09.012

[deae046-B26] Hoftiezer L , Hukkelhoven CW, Hogeveen M, Straatman HM, van Lingen RA. Defining small-for-gestational-age: prescriptive versus descriptive birthweight standards. Eur J Pediatr 2016;175:1047–1057. 27255904 10.1007/s00431-016-2740-8

[deae046-B27] IBM Corp. IBM SPSS Statistics for Windows. Armonk, NY: IBM Corp, 2021.

[deae046-B28] Jaddoe VW , de Jonge LL, Hofman A, Franco OH, Steegers EA, Gaillard R. First trimester fetal growth restriction and cardiovascular risk factors in school age children: population based cohort study. BMJ 2014;348:g14. 24458585 10.1136/bmj.g14 PMC3901421

[deae046-B29] Kalhan SC. One carbon metabolism in pregnancy: impact on maternal, fetal and neonatal health. Mol Cell Endocrinol 2016;435:48–60. 27267668 10.1016/j.mce.2016.06.006 PMC5014566

[deae046-B30] Korse CM , Buning-Kager J, Linders TC, Heijboer AC, van den Broek D, Tesselaar MET, van Tellingen O, van Rossum HH. A serum and platelet-rich plasma serotonin assay using liquid chromatography tandem mass spectrometry for monitoring of neuroendocrine tumor patients. Clin Chim Acta 2017;469:130–135. 28385629 10.1016/j.cca.2017.04.001

[deae046-B31] Laird NM , Ware JH. Random-effects models for longitudinal data. Biometrics 1982;38:963–974. 7168798

[deae046-B32] Lindström M , Tohmola N, Renkonen R, Hämäläinen E, Schalin-Jäntti C, Itkonen O. Comparison of serum serotonin and serum 5-HIAA LC-MS/MS assays in the diagnosis of serotonin producing neuroendocrine neoplasms: a pilot study. Clin Chim Acta 2018;482:78–83. 29596816 10.1016/j.cca.2018.03.030

[deae046-B33] Milner J , Arezina J. The accuracy of ultrasound estimation of fetal weight in comparison to birth weight: a systematic review. Ultrasound 2018;26:32–41. 29456580 10.1177/1742271X17732807 PMC5810856

[deae046-B34] Mondal A , Smith C, DuHadaway JB, Sutanto-Ward E, Prendergast GC, Bravo-Nuevo A, Muller AJ. IDO1 is an integral mediator of inflammatory neovascularization. EBioMedicine 2016;14:74–82. 27889479 10.1016/j.ebiom.2016.11.013 PMC5161421

[deae046-B35] Mook-Kanamori DO , Steegers EA, Eilers PH, Raat H, Hofman A, Jaddoe VW. Risk factors and outcomes associated with first-trimester fetal growth restriction. JAMA 2010;303:527–534. 20145229 10.1001/jama.2010.78

[deae046-B36] Most J , Dervis S, Haman F, Adamo KB, Redman LM. Energy intake requirements in pregnancy. Nutrients 2019;11:11. 10.3390/nu11081812 PMC6723706 31390778

[deae046-B37] Murphy MM , Higgins KA, Bi X, Barraj LM. Adequacy and sources of protein intake among pregnant women in the United States, NHANES 2003-2012. Nutrients 2021;13:795. 33670970 10.3390/nu13030795 PMC7997328

[deae046-B38] Oxenkrug GF. Metabolic syndrome, age-associated neuroendocrine disorders, and dysregulation of tryptophan-kynurenine metabolism. Ann N Y Acad Sci 2010;1199:1–14. 20633104 10.1111/j.1749-6632.2009.05356.x

[deae046-B39] Parisi F , Rousian M, Koning AH, Willemsen SP, Cetin I, Steegers-Theunissen RP. Periconceptional maternal one-carbon biomarkers are associated with embryonic development according to the Carnegie stages. Hum Reprod 2017;32:523–530. 28104698 10.1093/humrep/dew349

[deae046-B40] Pietersma CS , Mulders A, Sabanovic A, Willemsen SP, Jansen MS, Steegers EAP, Steegers-Theunissen RPM, Rousian M. The impact of maternal smoking on embryonic morphological development: the Rotterdam Periconception Cohort. Hum Reprod 2022;37:696–707. 35193145 10.1093/humrep/deac018 PMC8971648

[deae046-B41] R Core Team. R: A Language and Environment for Statistical Computing. Vienna, Austria: R Foundation for Statistical Computing, 2022.

[deae046-B42] Ranzil S , Ellery S, Walker DW, Vaillancourt C, Alfaidy N, Bonnin A, Borg A, Wallace EM, Ebeling PR, Erwich JJ et al Disrupted placental serotonin synthetic pathway and increased placental serotonin: potential implications in the pathogenesis of human fetal growth restriction. Placenta 2019;84:74–83. 31176514 10.1016/j.placenta.2019.05.012 PMC6724713

[deae046-B43] Rousian M , Koning AH, van Oppenraaij RH, Hop WC, Verwoerd-Dikkeboom CM, van der Spek PJ, Exalto N, Steegers EA. An innovative virtual reality technique for automated human embryonic volume measurements. Hum Reprod 2010;25:2210–2216. 20643693 10.1093/humrep/deq175

[deae046-B44] Rousian M , Schoenmakers S, Eggink AJ, Gootjes DV, Koning AH, Koster MP, Mulders AG, Baart EB, Reiss IK, Laven JS et al Cohort profile update: the Rotterdam periconceptional cohort and embryonic and fetal measurements using 3D ultrasound and virtual reality techniques. Int J Epidemiol 2021;50:1426–1427l. 34097026 10.1093/ije/dyab030 PMC8580268

[deae046-B45] Rubini E , Baijens IMM, Horánszky A, Schoenmakers S, Sinclair KD, Zana M, Dinnyés A, Steegers-Theunissen RPM, Rousian M. Maternal one-carbon metabolism during the periconceptional period and human foetal brain growth: a systematic review. Genes (Basel) 2021;12:1634. 34681028 10.3390/genes12101634 PMC8535925

[deae046-B46] Rubini E , Snoek KM, Schoenmakers S, Willemsen SP, Sinclair KD, Rousian M, Steegers-Theunissen RPM. First trimester maternal homocysteine and embryonic and fetal growth: the Rotterdam Periconception Cohort. Nutrients 2022;14:112. 10.3390/nu14061129 PMC8953595 35334786

[deae046-B47] Salas SP , Giacaman A, Romero W, Downey P, Aranda E, Mezzano D, Vío CP. Pregnant rats treated with a serotonin precursor have reduced fetal weight and lower plasma volume and kallikrein levels. Hypertension 2007;50:773–779. 17646571 10.1161/HYPERTENSIONAHA.107.094540

[deae046-B48] Schröcksnadel H , Baier-Bitterlich G, Dapunt O, Wachter H, Fuchs D. Decreased plasma tryptophan in pregnancy. Obstet Gynecol 1996;88:47–50. 8684760 10.1016/0029-7844(96)00084-1

[deae046-B49] Sha Q , Madaj Z, Keaton S, Escobar Galvis ML, Smart L, Krzyzanowski S, Fazleabas AT, Leach R, Postolache TT, Achtyes ED et al Cytokines and tryptophan metabolites can predict depressive symptoms in pregnancy. Transl Psychiatry 2022;12:35–38. 35078975 10.1038/s41398-022-01801-8 PMC8789799

[deae046-B50] Shabbir F , Patel A, Mattison C, Bose S, Krishnamohan R, Sweeney E, Sandhu S, Nel W, Rais A, Sandhu R et al Effect of diet on serotonergic neurotransmission in depression. Neurochem Int 2013;62:324–329. 23306210 10.1016/j.neuint.2012.12.014

[deae046-B51] Shibata K , Hirose J, Fukuwatari T. Method for evaluation of the requirements of B-group vitamins using tryptophan metabolites in human urine. Int J Tryptophan Res 2015;8:31–39. 25987848 10.4137/IJTR.S24412 PMC4404996

[deae046-B52] Smit AJP , Hojeij B, Rousian M, Schoenmakers S, Willemsen SP, Steegers-Theunissen RPM, van Rossem L. A high periconceptional maternal ultra-processed food consumption impairs embryonic growth: the Rotterdam periconceptional cohort. Clin Nutr 2022;41:1667–1675. 35772220 10.1016/j.clnu.2022.06.006

[deae046-B53] Steegers-Theunissen RP , Twigt J, Pestinger V, Sinclair KD. The periconceptional period, reproduction and long-term health of offspring: the importance of one-carbon metabolism. Hum Reprod Update 2013;19:640–655. 23959022 10.1093/humupd/dmt041

[deae046-B54] Steegers-Theunissen RP , Verheijden-Paulissen JJ, van Uitert EM, Wildhagen MF, Exalto N, Koning AH, Eggink AJ, Duvekot JJ, Laven JS, Tibboel D et al Cohort profile: the Rotterdam Periconceptional Cohort (Predict Study). Int J Epidemiol 2016;45:374–381. 26224071 10.1093/ije/dyv147

[deae046-B55] United Nations Educational Scientific and Cultural Organization. International Standard Classification of Education: ISCED 2011. Montreal: UNESCO Institute for Statistics, 2012, 85.

[deae046-B56] Ustunyurt E , Simsek H, Korkmaz B, Iskender C. First-trimester crown-rump length affects birth size symmetrically. J Matern Fetal Neonatal Med 2015;28:2070–2073. 25327173 10.3109/14767058.2014.978278

[deae046-B57] van Dijk MR , Borggreven NV, Willemsen SP, Koning AHJ, Steegers-Theunissen RPM, Koster MPH. Maternal lifestyle impairs embryonic growth: the Rotterdam Periconception Cohort. Reprod Sci 2018;25:916–922. 28884629 10.1177/1933719117728801

[deae046-B58] van Uitert EM , Exalto N, Burton GJ, Willemsen SP, Koning AH, Eilers PH, Laven JS, Steegers EA, Steegers-Theunissen RP. Human embryonic growth trajectories and associations with fetal growth and birthweight. Hum Reprod2013a;28:1753–1761. 23569080 10.1093/humrep/det115

[deae046-B59] van Uitert EM , van der Elst-Otte N, Wilbers JJ, Exalto N, Willemsen SP, Eilers PH, Koning AH, Steegers EA, Steegers-Theunissen RP. Periconception maternal characteristics and embryonic growth trajectories: the Rotterdam Predict study. Hum Reprod2013b;28:3188–3196. 24105824 10.1093/humrep/det375

[deae046-B60] van Zundert SK , Broekhuizen M, Smit AJ, van Rossem L, Mirzaian M, Willemsen SP, Danser AJ, De Rijke YB, Reiss IK, Merkus D et al The role of the kynurenine pathway in the (patho)physiology of maternal pregnancy and fetal outcomes: a systematic review. Int J Tryptophan Res2022a;15:11786469221135545. 36467775 10.1177/11786469221135545 PMC9716456

[deae046-B61] van Zundert SKM , Griffioen PH, van Rossem L, Willemsen SP, de Rijke YB, van Schaik RHN, Steegers-Theunissen RPM, Mirzaian M. Simultaneous quantification of tryptophan metabolites by liquid chromatography tandem mass spectrometry during early human pregnancy. Clin Chem Lab Med2022b;61:442–451. 36458576 10.1515/cclm-2022-0790

[deae046-B62] Wang Q , Zhang M, Ding Y, Wang Q, Zhang W, Song P, Zou MH. Activation of NAD(P)H oxidase by tryptophan-derived 3-hydroxykynurenine accelerates endothelial apoptosis and dysfunction in vivo. Circ Res 2014;114:480–492. 24281189 10.1161/CIRCRESAHA.114.302113 PMC4104160

[deae046-B63] Wang Y , Liu H, McKenzie G, Witting PK, Stasch JP, Hahn M, Changsirivathanathamrong D, Wu BJ, Ball HJ, Thomas SR et al Kynurenine is an endothelium-derived relaxing factor produced during inflammation. Nat Med 2010;16:279–285. 20190767 10.1038/nm.2092 PMC3556275

[deae046-B64] Worton SA , Pritchard HAT, Greenwood SL, Alakrawi M, Heazell AEP, Wareing M, Greenstein A, Myers JE. Kynurenine relaxes arteries of normotensive women and those with preeclampsia. Circ Res 2021;128:1679–1693. 33656370 10.1161/CIRCRESAHA.120.317612 PMC8154175

[deae046-B65] Wurtman RJ , Wurtman JJ, Regan MM, McDermott JM, Tsay RH, Breu JJ. Effects of normal meals rich in carbohydrates or proteins on plasma tryptophan and tyrosine ratios. Am J Clin Nutr 2003;77:128–132. 12499331 10.1093/ajcn/77.1.128

[deae046-B66] Xu Y , Ni M, Zhang Q, Zhao J, Tang Z, Liu Z. Correlation between crown-rump length in the first trimester of pregnancy and neonatal outcomes. BMC Pediatr 2022;22:386. 35778680 10.1186/s12887-022-03426-8 PMC9248167

[deae046-B67] Yaman S , Ceyhan M, Hancerliogullari N, Koc EM, Candar T, Tokmak A. Serum levels of kynurenine in pregnancies with fetal growth restriction and oligohydramnios. J Perinat Med 2023;51:641–645. 36586131 10.1515/jpm-2022-0446

[deae046-B68] Zhao YJ , Zhou C, Wei YY, Li HH, Lei W, Boeldt DS, Wang K, Zheng J. Differential distribution of tryptophan-metabolites in fetal and maternal circulations during normotensive and preeclamptic pregnancies. Reprod Sci 2022;29:1278–1286. 34622427 10.1007/s43032-021-00759-0 PMC8917071

