# Supplementary material for: First trimester maternal tryptophan metabolism and embryonic and fetal growth: the Rotterdam Periconceptional Cohort (Predict Study)
Source: Hum Reprod. 2024 Mar 18;39(5):912–22. doi: 10.1093/humrep/deae046 (PMC11063566; doi:10.1093/humrep/deae046)
Supplement: deae046_Supplementary_Table_S1 [file deae046_supplementary_table_s1.pdf]

OXFORD

human reproduction

Human Reproduction, 2024, 1–2
https://doi.org/10.1093/humrep/deae046
Supplementary Data

Supplementary Table S1. Periconceptional maternal baseline characteristics of the study sample used for the secondary analysis.

Baseline characteristics	Study sample secondary analysis ($n = 1433$)
**Periconceptional maternal characteristics**	
Age at conception (years)	32.2 (4.5)
Missing	*0*
BMI (kg/m^2)	25.7 (5.0)
Missing	*41*
Geographical background	
Western	1167 (86.2)
Non-Western	187 (13.8)
Missing	*79*
Educational level	
Low	93 (6.9)
Middle	475 (35.1)
High	785 (58.0)
Missing	*80*
Parity	
Nulliparous	669 (50.6)
Multiparous	654 (49.4)
Missing	*110*
Conception mode	
Natural	885 (61.8)
IVF/ICSI	548 (38.2)
Missing	*0*
Any smoking	
Yes	194 (14.3)
No	1158 (85.7)
Missing	*81*
Any alcohol use	
Yes	409 (30.3)
No	942 (69.7)
Missing	*82*
Any drug use	
Yes	20 (1.5)
No	1331 (98.5)
Missing	*82*
Folic acid supplement use[a]	
Adequate	1098 (81.3)
Inadequate	253 (18.7)
Missing	*82*
Energy intake (kJ/day)	8265.1 (2256.1)
Missing	*153*
Unreliable	*285*
Protein intake/energy intake (grams/day)	72.9 (19.4)
Missing	*153*
Unreliable	*285*
**Birth outcomes**	
Fetal sex	
Girl	683 (50.6)
Boy	668 (49.4)
Missing	*82*
EFW mid-pregnancy (grams)	352.0 (46.5)
Missing	*127*
EFW mid-pregnancy (z-score)	0.47 (1.1)
Missing	*133*
Gestational age at delivery (weeks)	38.9 (2.3)
Missing	*84*
Birthweight (grams)	3280.9 (587.6)
Missing	*93*
Birthweight (z-score)	–0.1 (1.1)
Missing	*104*

(continued)

Supplementary Table S1. Continued

Baseline characteristics	Study sample secondary analysis ($n = 1433$)
SGA (birthweight <p10)	
Yes	172 (12.0)
No	1157 (80.7)
Missing	*104*
**Tryptophan metabolites**	
TRP (μmol/l)	56.0 (9.7)<br>28.8–101.5
KYN (μmol/l)	1.5 (0.3)<br>0.7–3.45
5-HTP (nmol/l)	4.5 (1.7)<br>1.1–28.9
5-HT (nmol/l)	668.9 (283.1)<br>16.1–2180.2
5-HIAA (nmol/l)	38.6 (31.6–50.3)<br>15.9–538.7
Natural log of 5-HIAA (nmol/l)	3.8 (0.5)<br>2.8–6.3

[a] Folic acid supplement use was considered adequate when initiated before conception. Continuous data are presented as means with SD or median with interquartile range (5-HIAA), and categorical data as numbers with percentages. For the tryptophan metabolites also, the range (minimum–maximum) is given. 5-HIAA: 5-hydroxyindoleacetic acid; 5-HT: 5-hydroxytryptamine; 5-HTP: 5-hydroxytryptophan; AC: abdominal circumference; EFW: estimated fetal weight; KYN: kynurenine; TRP: tryptophan.
