# Supplementary material for: First trimester maternal tryptophan metabolism and embryonic and fetal growth: the Rotterdam Periconceptional Cohort (Predict Study)
Source: Hum Reprod. 2024 Mar 18;39(5):912–22. doi: 10.1093/humrep/deae046 (PMC11063566; doi:10.1093/humrep/deae046)
Supplement: deae046_Supplementary_Table_S2 [file deae046_supplementary_table_s2.pdf]

OXFORD
human reproduction
*Human Reproduction*, 2024, 1–1
https://doi.org/10.1093/humrep/deae046
Supplementary Data

**Supplementary Table S2.** Periconceptional maternal baseline characteristics of the included and excluded population.

| Baseline characteristics | Total study population (n = 1115) | Excluded population (n = 936) | Excluded population based on missing first trimester ultrasound or serum sample (n = 280) |
|---|---|---|---|
| Age at conception (years) | 32.5 (4.5) | 32.2 (4.8) | 32.0 (4.7) |
| *Missing* | *0* | *0* | *0* |
| BMI (kg/m$^2$) | 25.5 (4.8) | 26.3 (5.4) | 26.3 (5.9) |
| *Missing* | *30* | *43* | *8* |
| Geographical background | | | |
| Western | 918 (86.3) | 650 (84.4) | 157 (84.0) |
| Non-Western | 146 (13.7) | 120 (15.6) | 30 (16.0) |
| *Missing* | *51* | *166* | *93* |
| Educational level | | | |
| Low | 73 (6.9) | 74 (9.7) | 20 (10.9) |
| Middle | 366 (34.4) | 282 (36.9) | 59 (32.1) |
| High | 626 (58.8) | 409 (53.5) | 105 (57.1) |
| *Missing* | *50* | *171* | *96* |
| Parity | | | |
| Nulliparous | 597 (54.3) | 107 (43.0) | 107 (43.0) |
| Multiparous | 503 (45.7) | 142 (57.0) | 142 (57.0) |
| *Missing* | *15* | *31* | *31* |
| Conception mode[a] | | | |
| Natural | 567 (50.9) | 432 (80.9) | 24 (68.6) |
| IVF/ICSI | 548 (49.1) | 102 (19.1) | 11 (31.4) |
| *Missing* | *0* | *402* | *245* |
| Any smoking | | | |
| Yes | 147 (13.8) | 118 (15.6) | 30 (16.6) |
| No | 917 (86.2) | 639 (84.4) | 151 (83.4) |
| *Missing* | *51* | *179* | *99* |
| Any alcohol use | | | |
| Yes | 308 (29.0) | 224 (29.5) | 55 (30.2) |
| No | 755 (71.0) | 535 (70.5) | 127 (69.8) |
| *Missing* | *52* | *177* | *98* |
| Any drug use | | | |
| Yes | 15 (1.4) | 10 (1.3) | 1 (0.6) |
| No | 1049 (98.6) | 746 (97.7) | 180 (99.4) |
| *Missing* | *51* | *180* | *99* |
| Folic acid supplement use[b] | | | |
| Adequate | 896 (84.4) | 570 (74.7) | 134 (73.2) |
| Inadequate | 166 (15.6) | 193 (25.3) | 49 (26.8) |
| *Missing* | *53* | *173* | *97* |
| Energy intake (kJ/day) | 8215.0 (2223.2) | 8570 (2540) | 9080 (2270) |
| *Missing* | *108* | *260* | *110* |
| *Unreliable* | *216* | *150* | *31* |
| Protein intake/energy intake (grams/day) | 72.6 (19.6) | 74.3 (20.1) | 75.4 (21.1 |
| *Missing* | 108 | 260 | 110 |
| *Unreliable* | 216 | 150 | 31 |

[a] Conception mode was confirmed during the first trimester ultrasound appointment; therefore, among women without available first trimester ultrasound data, information on conception mode is also missing (n = 245).
[b] Folic acid supplement use was considered adequate when initiated before conception.
Continuous data are presented as mean with SD and categorical data as numbers with percentages.
