# Supplementary material for: First trimester maternal tryptophan metabolism and embryonic and fetal growth: the Rotterdam Periconceptional Cohort (Predict Study)
Source: Hum Reprod. 2024 Mar 18;39(5):912–22. doi: 10.1093/humrep/deae046 (PMC11063566; doi:10.1093/humrep/deae046)
Supplement: deae046_Supplementary_Table_S3 [file deae046_supplementary_table_s3.pdf]

OXFORD
human reproduction

Human Reproduction, 2024, 1–1
https://doi.org/10.1093/humrep/deae046
Supplementary Data

Supplementary Table S3. Associations between first trimester maternal tryptophan metabolites and standardized birthweight.

		BW (z-score)		
**Study sample secondary analysis (n = 1433)**		β	95% CI	
**TRP (μmol/l)**	Model 1	0.000	−0.006	0.006
	Model 2	0.002	−0.005	0.009
**KYN (μmol/l)**	Model 1	0.188*	0.010	0.365
	Model 2	0.064	−0.155	0.283
**5-HTP (nmol/l)**	Model 1	−0.008	−0.043	0.026
	Model 2	−0.025	−0.069	0.019
**5-HT (nmol/l)**	Model 1	0.000	0.000	0.000
	Model 2	0.000	−0.000	0.000
**Ln 5-HIAA (nmol/l)**	Model 1	0.238	−0.033	0.509
	Model 2	0.052	−0.087	0.191
**KYN/TRP ratio · 10^3**	Model 1	0.007	−0.001	0.016
	Model 2	−0.003	−0.014	0.008
**5-HTP/TRP ratio · 10^6**	Model 1	−0.681	−2.578	1.216
	Model 2	−0.002	−0.005	0.000

Model 1 is the crude model. Model 2 is adjusted for gestational age at the blood draw, maternal age, geographical origin, educational level, smoking, folic acid supplement use, protein intake/energy intake, BMI, conception mode, and parity.
5-HIAA: 5-hydroxyindoleacetic acid; 5-HT: 5-hydroxytryptamine; 5-HTP: 5-hydroxytryptophan; BW: birthweight; KYN: kynurenine; TRP: tryptophan.
* *P*-value ≤ 0.05 (statistically significant).
